# Gut microbiota-derived lipid metabolites facilitate regulatory T cell differentiation

**DOI:** 10.1038/s41598-023-35097-5

**Published:** 2023-06-01

**Authors:** Hiroaki Shiratori, Hiroyuki Oguchi, Yosuke Isobe, Kyu-Ho Han, Akira Sen, Kyosuke Yakebe, Daisuke Takahashi, Michihiro Fukushima, Makoto Arita, Koji Hase

**Affiliations:** 1 Division of Biochemistry, Department of Pharmaceutical Sciences, Faculty of Pharmacy, and Graduate School of Pharmaceutical Sciences, Keio University, Minato-ku, Tokyo, 105-8512 Japan; 2 Laboratory for Metabolomics, RIKEN Center for Integrative Medical Sciences (IMS), Yokohama, Kanagawa 230-0045 Japan; 3 Division of Physiological Chemistry and Metabolism, Graduate School of Pharmaceutical Sciences, Keio University, Minato-ku, Tokyo, 105-8512 Japan; 4 Department of Life and Food Sciences, Obihiro University of Agriculture and Veterinary Medicine, Obihiro, Hokkaido 080-8555 Japan; 5 The Institute of Fermentation Sciences (IFeS), Faculty of Food and Agricultural Sciences, Fukushima University, Kanayagawa, Fukushima, 960-1296 Japan; 6 International Research and Development Centre for Mucosal Vaccines, The Institute of Medical Science, The University of Tokyo (IMSUT), Minato-ku, Tokyo, 108-8639 Japan

**Keywords:** Lipidomics, Mucosal immunology

## Abstract

Commensal bacteria-derived metabolites are critical in regulating the host immune system. Although the impact of gut microbiota-derived hydrophilic metabolites, such as short-chain fatty acids, on immune cell functions and development has been well documented, the immunomodulatory effects of gut microbiota-derived lipids are still of interest. Here, we report that lipid extracts from the feces of specific-pathogen-free (SPF), but not germ-free (GF), mice showed regulatory T (Treg)-cell-inducing activity. We conducted RP-HPLC-based fractionation and liquid chromatography–tandem mass spectrometry (LC–MS/MS)-based lipidome profiling and identified two bioactive lipids, 9,10-dihydroxy-12Z-octadecenoic acid (9,10-DiHOME) and all-*trans* retinoic acid (atRA), with Treg-inducing activity in vitro. The luminal abundance of 9,10-DiHOME in the large intestine was significantly decreased by dextran sulfate sodium (DSS)-induced colitis, indicating that 9,10-DiHOME may be a potential biomarker of colitis. These observations implied that commensal bacteria-derived lipophilic metabolites might contribute to Treg development in the large intestine.

## Introduction

The intestinal mucosa is constantly exposed to foreign substances and thus possesses several lines of defense mechanisms, including the epithelial barrier and secretory IgA. Meanwhile, harmless substances, such as food antigens and gut microbiota-produced antigens, exist in the intestinal mucosa. However, exaggerated immune responses to these innocuous antigens often cause intestinal disorders (e.g., celiac disease, food allergies, and inflammatory bowel disease). Therefore, the intestinal immune system elicits immune tolerance against harmless foreign antigens, termed mucosal tolerance.

Regulatory T (Treg) cells play vital roles in inducing mucosal tolerance. Treg cells express forkhead box P3 (Foxp3) as a master transcription factor and suppress the immune response to harmless foreign antigens. Treg cells inhibit the activation of surrounding effector T cells by expressing a high-affinity interleukin-2 (IL-2) receptor, CD25, and by producing suppressor cytokines, such as IL-10 and transforming growth factor-β1 (TGF-β1)^1^. Treg cells also express cytotoxic T-lymphocyte-associated protein 4 (CTLA-4), an inhibitory co-receptor that binds to B7 molecules on the surface of antigen-presenting cells, such as dendritic cells (DCs), to suppress their activation. Mice lacking the CNS1 enhancer region of the *Foxp3* gene locus are nearly deficient in peripherally induced Treg (pTreg) cells and spontaneously develop allergic-like bronchitis owing to an increase in Th2 cells^2^. Treg cells exist in tissues throughout the body and comprise approximately 10% of the total CD4^+^ T cells in most peripheral tissues. However, the frequency of Treg cells is much higher (approximately 30%) in the colon^3^. Compelling evidence has revealed that colonization by commensal microbiota facilitates the development of colonic Treg cells^4^. Under germ-free (GF) conditions, the frequency of Treg cells among colonic CD4^+^ T cells was reduced to approximately 10%. Treg cells induced by commensal bacteria express the transcription factor RAR-related orphan receptor γ (RORγt), which was previously thought to be specifically expressed in IL-17-producing Th17 cells. In mice lacking Treg cell-specific RORγt (Foxp3^Cre^ RORγt^F/F^), the number of Th2 cells in the intestine is significantly increased, and oxazolone-induced colitis is exacerbated^5^. Furthermore, we found that T cell-specific Uhrf1 deficiency impairs the expansion and functional maturation of intestinal Treg cells, leading to the spontaneous development of severe colitis^6^. These observations illustrate the biological significance of Treg cells in maintaining immune homeostasis in mucosal tissue.

Early studies demonstrated that bacterial components and metabolites from certain commensal bacteria are responsible for the induction of pTreg cells. For instance, it was previously found that a fermented product, butyrate, derived from the bacterial consortium belonging to *Clostridium* cluster IV and XIVa, facilitates Treg cell differentiation both in vitro and in vivo^7^. In contrast, peptidoglycan, a major cell membrane component of *Clostridium butyricum*, increases TGF-β production by CD103^+^ DCs and IL-10 production by macrophages by stimulating Toll-like receptor 2 (TLR2), thereby inducing Treg cell differentiation in the colon^8^. Moreover, macrophages in the colonic lamina propria release IL-1β via the activation of TLR signaling by commensal bacteria, which promotes the production of granulocyte–macrophage colony-stimulating factor (GM-CSF) from type 3 innate lymphoid cells (ILC3). GM-CSF produced by ILC3 promotes Treg cell differentiation in the colonic lamina propria and mesenteric lymph node (MLN) by increasing the production of IL-10 and retinoic acid by DCs and macrophages^9^. *Bacteroides fragilis* promotes IL-10 production by T cells by producing polysaccharide A (PSA)^10^. *Bacteroides distasonis*, *Lactobacillus murinus*, *Lactobacillus acidophilus*, and *Mucispirillum schaedleri* have been identified as bacteria that induce Treg cell differentiation, although the underlying mechanism is yet to be clarified^11^. Thus, commensal bacteria promote Treg cell development via multiple mechanisms.

Multiple lines of evidence have demonstrated that the gut microbiota utilizes dietary polyunsaturated fatty acid (PUFA) as a substrate to produce a variety of lipophilic metabolites, which affect host physiology, through oxidation, hydroxylation, and saturation^12^. Some lipophilic metabolites derived from commensal bacteria have anti-inflammatory properties. For example, 10-hydroxy-*cis*-12-octadecenoic acid (HYA), a derivative of linoleic acid (LA) produced by *Lactobacillus plantarum*, suppresses the maturation of DCs and attenuates the production of inflammatory cytokines^13^. Furthermore, 13-hydroxy-9(Z),15(Z)-octadecadienoic acid and 13-oxo-9(Z),15(Z)-octadecadienoic acid, which are produced from α-linolenic acid by lactic acid bacteria, induce the differentiation of anti-inflammatory M2 macrophages^14^. These findings suggest that fatty acid metabolism by commensal bacteria regulates host immunity by regulating macrophage differentiation. However, the effects of gut microbiota-derived lipophilic metabolites (except secondary bile acids) on other immune cell populations, including T cells, remain undetermined. Here, we demonstrate that the lipid extract of mouse feces had Treg cell-inducing activity in vitro and identified 9,10-dihydroxy-12Z-octadecenoic acid (9,10-DiHOME) and all-*trans* retinoic acid (atRA) as active components by liquid chromatography–tandem mass spectrometry (LC–MS/MS)-based lipidomics and RP-HPLC-based fractionation.

## Results

### Gut microbiota-derived lipophilic metabolites induce Treg cell differentiation in vitro

**Figure 1:**
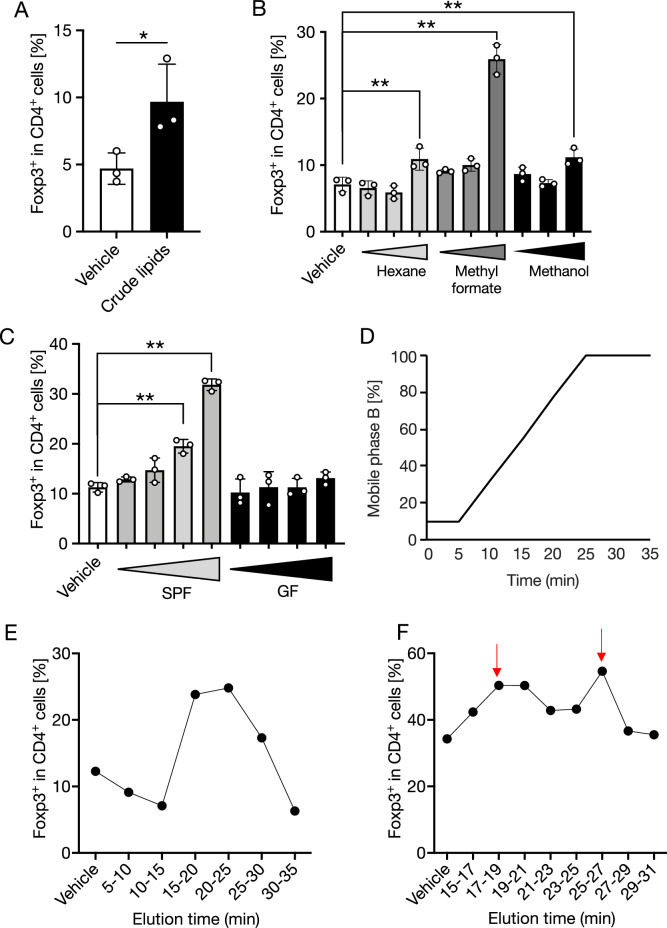
Lipids extracted from SPF mouse feces facilitate Treg differentiation in vitro. (**A**–**C**) Splenic naïve T cells and DCs were co-cultured in the presence of crude lipids (**A**), hexane, methyl formate, and methanol fractions obtained by solid-phase extraction (**B**), and methyl formate fraction obtained by solid-phase extraction from feces of SPF mice and GF mice (**C**). The frequency of Foxp3^+^ Treg cells in the CD4^+^ TCRβ^+^ T cells gate was examined by flow cytometry on day 3 of culture. (**D**) HPLC used a C18 column with a gradient of water/methanol/acetic acid (50:50:0.01, v/v/v, mobile phase A) and methanol (mobile phase B). (**E**,**F**) The lipids of the methyl formate fraction from SPF mouse feces were fractionated by HPLC every (**E**) 5 min or (**F**) 2 min. Each fraction was diluted 6400-, 1600-, or 400-fold (**B**) and 1600-, 800-, 400-, or 200-fold (**C**) and added to the medium. Treg-inducing ability of each fraction was evaluated by flow cytometry. The fractions indicated by red arrows in (**F**) displayed higher Treg-inducing activity. The data represent the mean ± SD (**A**–**C**,**E** *n* = 3, **F** *n* = 2) *P < 0.05, **P < 0.01, *P* values were determined by unpaired *t*-test (**A**) and one-way ANOVA followed by Dunnett's test (**B**,**C**,**E**). *DCs* dendritic cells, *SPF* specific-pathogen-free, *GF* germ-free, *HPLC* high-performance liquid chromatography.

To examine whether fecal lipophilic metabolites promote Treg cell differentiation, splenic DCs and naïve T cells were co-cultured for three days under Treg-skewing conditions with crude methanol extract prepared from the feces of specific-pathogen-free (SPF) mice (Supplemental Fig. 1A). The crude methanol extract significantly increased the proportion of Foxp3^+^ Treg cells compared to that with the vehicle control (Fig. 1A).

To determine the active constituents, we first separated the crude methanol extract into three fractions of different lipophilicities using a solid-phase extraction method. Hexane, methyl formate, and methanol were passed through a C18 column, on which the crude methanol extract was absorbed. We subsequently evaluated the Treg-inducing effect of each fraction in a co-culture system. Among these, the methyl formate fraction exhibited the highest Treg cell-inducing activity (Fig. 1B). In contrast, the methyl formate fraction prepared from GF mouse feces showed no effect on Treg cell induction (Fig. 1C). LC–MS/MS-based lipidomics of methyl formate fractions showed that various PUFAs, such as eicosapentaenoic acid, docosahexaenoic acid, LA, and arachidonic acid, were significantly reduced in the feces of GF mice (Supplemental Fig. 2A–E). We fractionated the methyl-formate by polarity using high-performance liquid chromatography (HPLC). The HPLC system used a C18 column with a gradient of water/methanol/acetic acid (50:50:0.01, v/v/v, mobile phase A) and methanol (mobile phase B) (Fig. 1D). The objective of fractioning was to identify the metabolites responsible for promoting Treg cell differentiation. We collected the fractions at 5-min intervals and then assessed Treg-inducing activity. The 15–20, 20–25, and 25–30 min eluting fractions significantly promoted Treg differentiation (Fig. 1E). Because of the high Treg cell-inducing activity of the 15–30 min eluate, we further separated this eluate mixture at 2-min intervals. We detected Treg cell-inducing activity in the eluate at 17–19 min and 25–27 min (Fig. 1F).

### 9,10-DiHOME is an active constituent in the 17–19 min-eluted fraction

**Figure 2:**
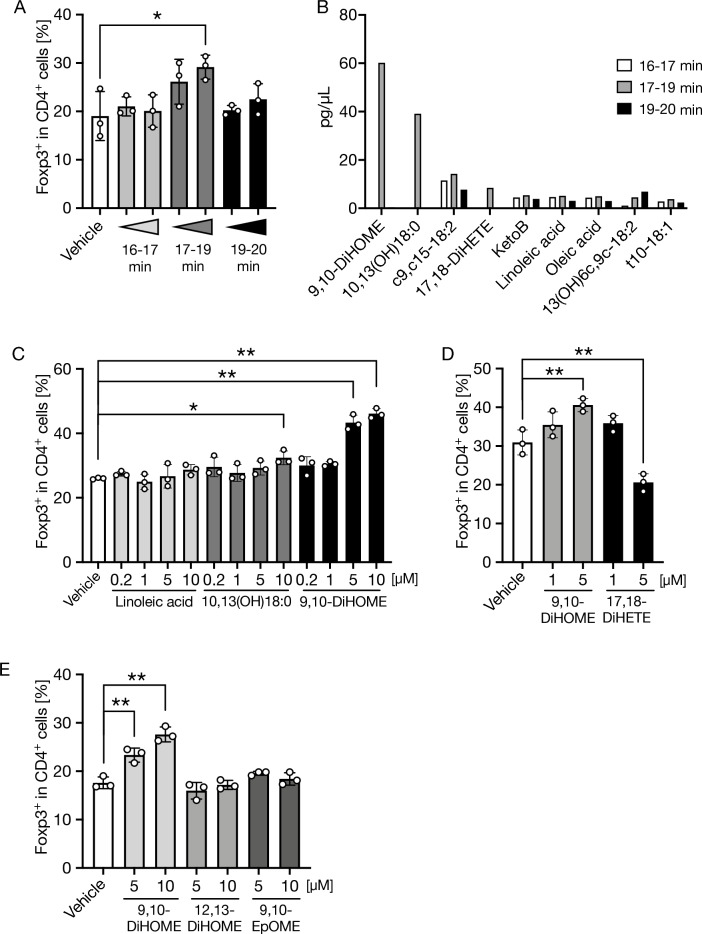
9,10-DiHOME in 17–19 min fraction facilitates Treg differentiation in vitro. Methyl formate fraction was extracted from SPF mouse feces, and the 16–20 min elution fraction was collected by HPLC, followed by LC–MS/MS targeted lipidomics for oxidized fatty acids and evaluation of Treg-inducing ability by flow cytometry. (**A**,**C**–**E**) The frequency of Foxp3^+^ Treg cells in CD4^+^ TCRβ^+^ cells gate after 3-day culture of naïve T cells in the presence of 16–17, 17–19, and 19–20 min fractions of the methyl formate extract (**A**); LA, 9,10-DiHOME, 10,13(OH)18:0, and 17,18-DiHETE (**C**,**D**); and 9,10-DiHOME, 12,13-DiHOME, and 9,10-EpOME (**E**) was analyzed by flow cytometry. (**A**) Each fraction was diluted 800- or 200-fold and added to the medium. (**B**) Targeted lipidomics analysis of 16–17, 17–19, and 19–20 min of fractions of the methyl formate extract. The data represent the mean ± SD. *P < 0.05, **P < 0.01 (**A**,**C**–**E** *n* = 3, **B** *n* = 1). *P* values were determined by one-way ANOVA followed by Dunnett's test (**A**,**C**–**E**). *SPF* specific-pathogen-free, *HPLC* high-performance liquid chromatography, *LC–MS/MS* liquid chromatography–tandem mass spectrometry, *LA* linoleic acid, *DiHOME* dihydroxy-12Z-octadecenoic acid, *DiHETE* dihydroxy-eicosa-5,8,11,14-tetraenoic acid, *EpOME* 9,10-epoxy-12Z-octadecenoic acid, *10,13(OH)18:0* 10,13-dihydroxy-18:0

The methyl formate fraction is widely used for analysis of oxidized fatty acids^15, 16^. To identify candidate lipid metabolites in the 17–19 min eluate, we performed comprehensive and quantitative lipidomics of fatty acid derivatives using LC–MS/MS^17^. We analyzed the 16–17 and 19–20 min eluates as negative references, which lacked Treg-inducing activity (Fig. 2A). Lipidomics analysis demonstrated that three oxidized fatty acids, 9,10-DiHOME, 10,13-dihydroxy-18:0 (10,13(OH)18:0), and 17,18-dihydroxy-eicosa-5,8,11,14-tetraenoic acid (17,18-DiHETE), were specifically contained in the 17–19 min fraction (Fig. 2B). We subsequently evaluated the Treg cell-inducing effects of these hydroxy fatty acids, with LA (a source of 9,10-DiHOME and 10,13(OH)18:0) as a negative control. Among these, only 9,10-DiHOME promoted Treg cell differentiation in a concentration-dependent manner (Fig. 2C, D). To further investigate the structure–activity correlation, we compared the Treg-inducing activity of 9,10-DiHOME, 12,13-DiHOME (a structural isomer of 9,10-DiHOME), and 9,10-epoxy-12Z-octadecenoic acid (9,10-EpOME, a precursor of 9,10-DiHOME). Notably, 9,10-DiHOME alone showed this differentiating activity, indicating that the Treg-inducing effect may be specific to 9,10-DiHOME among the structurally related oxidized fatty acids (Fig. 2E). Thus, we conclude that 9,10-DiHOME is a bioactive lipophilic metabolite in the 17–19 min fraction.

### atRA is one of the Treg cell-inducing lipid metabolites in 25–27 min fraction

**Figure 3:**
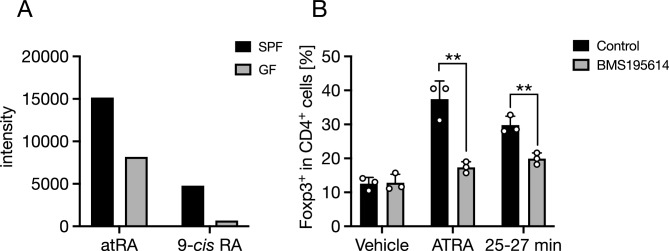
atRA in 25–27 min fraction of the methyl formate extract facilitates Treg differentiation in vitro. (**A**) Fecal concentrations of atRA and 9-*cis* RA of SPF and GF mice were analyzed by LC–MS/MS. (**B**) The frequency of Foxp3^+^ Treg cells in CD4^+^ TCRβ^+^ cells gate after 3-day culture with 25–27 min fraction of the methyl formate extract (Fig. 1F) and atRA in the presence or absence of BMS195614 was evaluated by flow cytometry. The data represent the mean ± SD. *P < 0.05, **P < 0.01 (**A** *n* = 1, **B** *n* = 3). *P* values were determined by two-way ANOVA followed by Šídák's multiple comparisons test (**B**). *atRA* all-*trans* retinoic acid, *9-cis RA* 9-*cis* retinoic acid, *SPF* specific-pathogen-free, *GF* germ-free, *LC–MS/MS* liquid chromatography–tandem mass spectrometry.

Next, we sought to identify the active constituents in the 25–27 min eluate fraction by comprehensive and quantitative lipidomic analysis using LC–MS/MS. atRA, a well-characterized Treg inducer, was present in this fraction. This fraction also contained 9-*cis* retinoic acid (9-*cis* RA). Notably, the fecal concentrations of both retinoic acids were much higher in SPF mice than in GF mice (Fig. 3A), implying that the gut microbes may provide atRA and 9-*cis* RA. atRA activates the retinoic acid receptor (RAR) to upregulate the expression of Foxp3, a master transcription factor in Treg cells^18, 19^. Therefore, we investigated whether inhibition of RAR signaling by BMS-195614^20^ affected the Treg-inducing activity of the 25–27 min eluate. BMS-195614 treatment at least partly abrogated the 25–27 min eluate-dependent Treg cell differentiation (Fig. 3B). Thus, atRA is most likely a principal bioactive constituent responsible for Treg induction in this fraction.

### 9,10-DiHOME induces Treg cell differentiation in the presence of DCs

We have previously used a co-culture system of naïve T cells and DCs to evaluate Treg-inducing activity. To examine whether 9,10-DiHOME acts directly on naïve T cells to promote Treg cell differentiation, splenic naïve T cells were cultured under Treg-skewing conditions in the presence or absence of 9,10-DiHOME without DCs. We observed that 9,10-DiHOME treatment only slightly promoted Treg differentiation at a high concentration (5 µM) (Supplemental Fig. 3A,B), suggesting that 9,10-DiHOME modulates the functions of DCs to facilitate Treg cell differentiation. Endogenous production of atRA by DCs is essential for Treg cell differentiation in vivo^21^. For instance, CD103^+^ DCs in the intestinal mucosa express retinal dehydrogenase 2 (RALDH2), a rate-limiting enzyme in atRA production, and thus promote Treg cell differentiation^22^. Bone marrow-derived DCs (BMDCs) induced by GM-CSF also express *Aldh1a2* and CCR9, functional markers characteristic of intestinal DCs^23^. Therefore, we analyzed RALDH activity in BMDCs treated with 9,10-DiHOME using an ALDEFLUOR assay. However, the treatment of BMDCs with 9,10-DiHOME did not influence RALDH activity (Supplemental Fig. 3C). Collectively, 9,10-DiHOME most likely promoted Treg differentiation through DCs in an atRA-independent manner.

### Gut microbes produce 9,10-DiHOME by oxidizing LA

**Figure 4:**
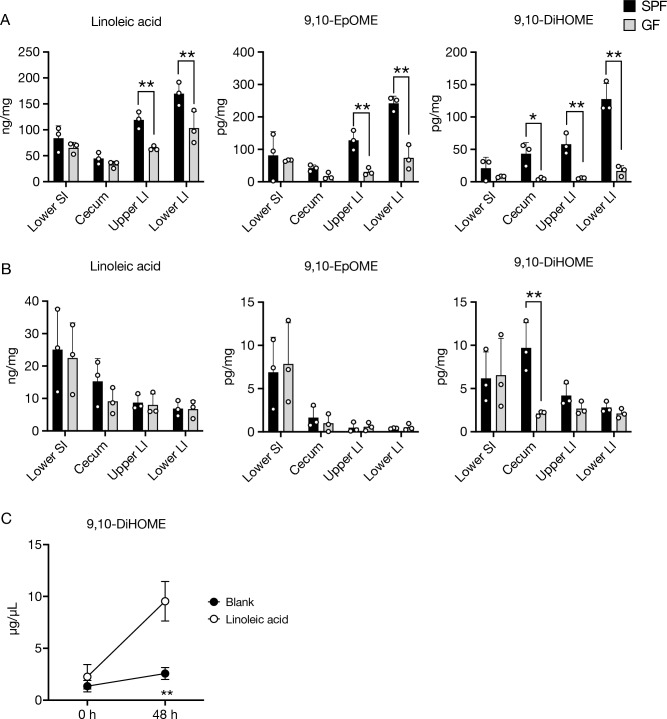
Gut microbes produce 9,10-DiHOME by oxidizing LA. (**A**,**B**) Concentrations of 9,10-DiHOME and precursors (LA and 9,10-EpOME) in the luminal contents in the colon (**A**) and colon tissues (**B**) of SPF and GF mice were analyzed by LC–MS/MS. (**C**) Rat cecal contents were cultured in ex vivo fermentation system in the presence or absence of LA. Concentrations of 9,10-DiHOME in the supernatants at 0 h and 48 h after addition of LA were analyzed by LC–MS/MS. The data represent the mean ± SD. *P < 0.05, **P < 0.01, *n* = 3. *P* values were determined by two-way ANOVA followed by Šídák's multiple comparisons test. *SI* small intestine, *LI* large intestine, *9,10-DiHOME* 9,10-dihydroxy-12Z-octadecenoic acid, *LA* linoleic acid, *9,10-EpOME* 9,10-epoxy-12Z-octadecenoic acid, *SPF* specific-pathogen-free, *GF* germ-free.

9,10-DiHOME is a metabolite of LA. LA is initially converted to 9,10-EpOME and then to 9,10-DiHOME by cytochrome P450 epoxygenases and epoxide hydrolase, respectively^24, 25^. To assess whether commensal bacteria produce 9,10-DiHOME, we compared the concentrations of 9,10-DiHOME and its precursors in the intestinal tissues and intestinal contents of SPF and GF mice. The concentration of 9,10-DiHOME in the intestinal contents of the cecum and colon was much lower in GF mice than in SPF mice (Fig. 4A). A similar trend was observed in the analysis of 9,10-EpOME. Likewise, a reduction in 9,10-DiHOME concentration in the cecal tissue of GF mice was evident. However, there was no significant difference in the values of the colon and small intestine (Fig. 4B). These data indicate that the gut microbes contribute, at least partially, to the production of 9,10-EpOME and 9,10-DiHOME. To further confirm the contribution of the gut microbes to the generation of 9,10-DiHOME, we cultured rat cecal microbiota in the presence or absence of LA, using an ex vivo fermentation system^26^. Supplementation of LA increased the concentration of 9,10-DiHOME in the culture medium within 48 h (Fig. 4C). Thus, we conclude that the gut microbes substantially metabolize LA to 9,10-DiHOME.

### The effect of 9,10-DiHOME on Treg cells in vivo

We subsequently examined whether 9,10-DiHOME induces Treg cells in vivo. The mice received intragastric gavage of 9,10-DiHOME. However, 9,10-DiHOME did not change the number of Treg cells in the small intestine and colon, and MLN (Supplemental Fig. 4A). Furthermore, intrarectal or intraperitoneal administration of 9,10-DiHOME also did not affect the abundance of Treg cells (Supplemental Fig. 4B,C). Thus, exogenous administration of 9,10-DiHOME failed to induce Treg cell expansion in vivo.

### Intestinal inflammation decreases the luminal amount of 9,10-DiHOME

**Figure 5:**
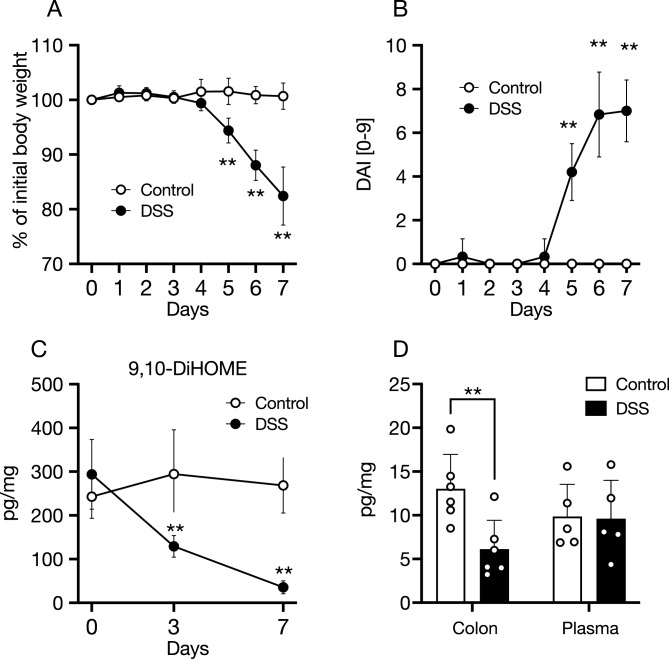
9,10-DiHOME is decreased with the exacerbation of DSS colitis. Mice were treated with 2% DSS or RO water for 7 days, and 9,10-DiHOME concentrations in feces, colon tissue, and plasma were measured by LC–MS/MS. (**A**,**B**) Body weight change (**A**) and DAI (**B**) of 2% DSS- or RO water-treated mice during 7 days of study. DAI score was calculated by body weight loss, stool consistency, and stool bleeding. (**C**) Concentration of 9,10-DiHOME in feces from day 0 to day 7 after DSS administration. (**D**) Concentration of 9,10-DiHOME in colon tissue and plasma on day 7 after DSS administration. The data represent the mean ± SD. *P < 0.05, **P < 0.01. *P* values were determined by Student's *t*-tests (**A**–**C**) and two-way ANOVA followed by Šídák's multiple comparisons test (**D**). *9,10-DiHOME* 9,10-dihydroxy-12Z-octadecenoic acid, *DSS* dextran sulfate sodium, *RO* reverse osmosis, *DAI* disease activity index.

We further investigated the production of 9,10-DiHOME under inflammation conditions. We administered dextran sulfate sodium (DSS) in drinking water to mice (Fig. 5A,B) and analyzed the amount of 9,10-DiHOME in the feces, colonic tissue, and plasma by LC–MS/MS. We observed that 9,10-DiHOME in the feces decreased significantly from day three onwards (Fig. 5C). The reduction in luminal 9,10-DiHOME preceded the exacerbation of colitis, as indicated by body weight loss and disease activity index (DAI) (Fig. 5A,B). Consistent with this observation, the amount of 9,10-DiHOME in colonic tissue also decreased on day 7 (Fig. 5D). In contrast, no significant differences were observed in the plasma levels of 9,10-DiHOME (Fig. 5D). These data suggest that intestinal inflammation impairs the microbial production of 9,10-DiHOME.

## Discussion

In this study, we found that the gut microbiota-derived lipophilic metabolites induce Treg differentiation. Multiple evidence has demonstrated that the gut microbiota-derived hydrophilic metabolites play a key role in immune homeostasis in the intestine^7^ and extraintestinal tissue^27, 28^. Meanwhile, our results demonstrated that the lipid extract from the feces of SPF, but not GF, mice manifested high Treg-inducing activity, implying the contribution of lipophilic metabolites to immune regulation by the gut microbiota.

We detected Treg-inducing activity in the methyl formate fraction of the fecal lipid extract. A previous study demonstrated that the methyl formate fraction contains abundant PUFA-derived metabolites and hydroxy fatty acids^16^. Indeed, our target lipidomics analysis identified 9,10-DiHOME and atRA as the bioactive constituents with Treg-inducing activity^29^. 9,10-DiHOME is produced by the cleavage of 9,10-EpOME, an LA derivative^30^, mediated by epoxide hydrolase. We observed that the luminal concentration of 9,10-DiHOME was significantly decreased under GF conditions. Furthermore, 9,10-DiHOME was generated by the addition of LA to the ex vivo fermentation system. Thus, the the gut microbes are responsible for the production of 9,10-DiHOME. In support of this view, early studies have shown that the gut microbiota contribute to the oxidation of LA^12, 31^. In particular, *Bifidobacterium bifidum* and *Enterococcus faecalis* are known to possess epoxide hydrolase to produce 12,13-DiHOME, a structural isomer of 9,10-DiHOME^25^. Although the major producers of 9,10-DiHOME remain unknown, these bacterial species may contribute to the generation of 9,10-DiHOME.

While the Treg-inducing effect of 9,10-DiHOME was obvious in the co-culture system with naïve T cells and DCs, its activity was substantially diminished under the monoculture conditions with naïve T cells. Therefore, 9,10-DiHOME may induce Treg cells indirectly through functional modifications of DCs. 9,10-DiHOME functions as an agonist of peroxisome proliferator-activated receptor gamma (PPARγ)^32^. Notably, activation of PPARγ in DCs leads to upregulation of *Aldh1a2* (also known as RALDH2), thereby promoting Treg differentiation through the production of atRA^33^. However, this pathway is unlikely to contribute to the Treg-inducing activity of 9,10-DiHOME because treatment with 9,10-DiHOME showed negligible effects on RALDH activity in DCs. Further investigations are required to elucidate the underlying mechanism by which 9,10-DiHOME confers Treg-inducing activity on DCs.

In this study, we also analyzed the Treg-inducing activity of 9,10-DiHOME precursors (LA and 9,10-EpOME) and a structure isomer (12,13-DiHOME); however, none of these compounds promoted Treg differentiation. Conversely, a previous report demonstrated that 12,13-DiHOME exacerbated allergic airway inflammation by inhibiting Treg differentiation, while 9,10-DiHOME had no effect on Treg differentiation^25^. This apparent discrepancy may be due to the differences in the experimental conditions between the previous study and ours. While the previous work used human monocyte-derived DCs to induce Treg cells, we employed mouse BMDCs. In addition, the concentration of 9,10-DiHOME used in the previous study was substantially higher (75–200 µM) than that used in the current study (1–10 µM). Thus, despite some dissenting views, our results suggest that the Treg-inducing effect of 9,10-DiHOME is a structure-specific property that is not common among LA metabolites.

We have examined the effects of exogenous 9,10-DiHOME administration on Treg differentiation in vivo. However, 9,10-DiHOME exhibited no Treg-inducing effect by any administration route in any tissue we tested. There is a possibility of the preexistence of a certain amount of 9,10-DiHOME in the intestine at a steady state. Like the bacteria, the mammalians also produce 9,10-DiHOME by expressing cytochrome P450 and epoxide hydrolase^34^. Indeed, the amount of 9,10-DiHOME in the intestinal tissue except the cecum remained unchanged even under GF conditions, although luminal amounts of 9,10-DiHOME was markedly decreased. We, therefore, speculate that the preexistence of 9,10-DiHOME may have prevented any additional Treg-inducing effect upon exogenous administration of 9,10-DiHOME, although further investigation will be needed to validate this speculation.

The concentration of 9,10-DiHOME in the feces and colonic tissue was reduced during the progression of DSS-induced colitis. Intestinal inflammation alters the microbial community, leading to the underrepresentation of obligate anaerobic bacteria and the overrepresentation of facultative anaerobic bacteria belonging to the family Enterobacteriaceae (e.g., *Escherichia coli*) that utilizes oxygen and nitrogen species generated under inflammatory conditions^35^. Such alterations in the microbial community may result in the underrepresentation of 9,10-DiHOME producers. Notably, a decrease in 9,10-DiHOME levels was observed at the early disease stage before the emergence of clinical symptoms. Therefore, although this possibility should be evaluated in human patients with inflammatory bowel disease, the fecal concentration of 9,10-DiHOME could serve as an early biomarker of colitis. atRA and 9-*cis*-RA were abundantly detected in the feces of SPF mice. Given that the fecal amount of retinoic acids was prominently reduced under GF conditions, the gut microbiota may also contribute to producing these retinoic acids. atRA and 9-*cis*-RA bind to the RARα and retinoid X receptor (RXR), respectively. The RARα/RXR heterodimer binds to the CNS1 enhancer region of the *Foxp3* gene locus to facilitate gene expression, leading to the induction of pTreg cells^2^. atRA is synthesized from dietary vitamin A (also known as retinol) through oxidative reactions catalyzed by retinol dehydrogenase and RALDH. In the intestine, CD103^+^ DCs, stromal cells, and enterocytes take up dietary vitamin A to produce atRA^29, 36–38^. In addition, segmented filamentous bacteria (SFB) express RALDH and produce retinoic acids^39^. Considering that SFB colonize the ileum but not the colon^40^, other bacterial species are most likely responsible for retinoic acid production in the colon.

In conclusion, we identified 9,10-DiHOME and atRA as gut microbiota-derived lipophilic metabolites with Treg-inducing activity. Our findings provide a new perspective regarding the importance of lipophilic metabolites derived from the gut microbiota in maintaining immune homeostasis in the intestine. Furthermore, we established an experimental platform to identify immunomodulatory lipophilic metabolites by combining HPLC fractionation with an in vitro Treg cell culture assay, followed by targeted lipidomics. Our observations suggest that this platform has potential in the discovery of additional biologically active lipophilic metabolites in the gut lumen.

## Methods

### Animals

All animal experiments were performed using protocols approved by the Animal Studies Committee of the RIKEN Yokohama Institute, Keio University, and Obihiro University of Agriculture and Veterinary Medicine. C57BL/6J mice were purchased from CLEA Japan (Tokyo, Japan) or Sankyo Labo Service Corporation (Tokyo, Japan). Mice were fed a normal chow diet (CE-2) (CLEA, Japan). GF IQI mice (CLEA Japan) were bred and maintained in GF isolators at the animal facility at Keio University. All GF mice were fed a sterilized normal chow diet (CMF) (Oriental Yeast, Tokyo, Japan). All mice were between 5 and 15 weeks of age at the onset of the experiments. Wister rats were obtained from Charles River Laboratories Japan (Yokohama, Japan). For dissection, the mice were euthanized by cervical dislocation after inhalation of excessive amounts of isoflurane.

In the separate experiment shown in Supplemental Fig. 4, 3-week-old mice obtained from CLEA Japan received 9,10-DiHOME (1–1000 µg/mouse) solubilized in 4% dimethyl sulfoxide every day for 3 weeks by intragastric administration, intrarectal administration, or intraperitoneal injection to analyze abundance of Treg cells in the small intestine, colon, spleen, and MLN.

### Lipid extraction

Lipid metabolites in the feces, intestinal contents, and tissues were extracted using solid-phase extraction. Briefly, 100 mg of mouse feces was homogenized twice in 1 mL of methanol using a bead homogenizer (PRECELLYS 24-DUAL, Bertin Technologies) at 6500 rpm for 15 s. The homogenized feces were placed at − 30 °C overnight to extract lipids. The crude methanol extract was aliquoted for bioassay, dried under a stream of nitrogen gas (N2 SUPPLIER MODEL 05BL, Sic System Instruments), and then dissolved in ethanol (1 µL/mg feces). The extract was then diluted 1:1600 with culture medium to determine Treg cell-induction activity. For further fractionation of crude methanol extracts by solid-phase extraction, the extracted lipids were diluted, adjusted to pH 3 with HCl, and injected into C18 Sep-Pak cartridges. After washing the sample trapped in the column with ultrapure water, the lipids were eluted sequentially in 10 mL each of hexane, methyl formate, and methanol. The collected samples were dried under a stream of nitrogen gas (N2 SUPPLIER MODEL 05BL, Sic System Instruments) and then dissolved in 100 µL of ethanol for bioassay or methanol for further HPLC fractionation.

The following methods were used to determine lipid metabolite concentrations in the feces, tissues, plasma, and bacterial culture supernatants. The tissue or feces sample was placed in a crush tube and crushed twice with a multi-specimen cell crusher (Multi-Shocker, Yasui Kikai) at 2500 rpm for 15 s. Lipids were extracted by adding ice-cold methanol, vortexing, and incubating overnight at − 30 °C. The extracted lipids were centrifuged at 4 °C, 3000 rpm for 10 min, and the supernatant was collected. Plasma and enterobacterial culture supernatants were vortexed with twice the volume of methanol and incubated overnight at − 30 °C to extract the lipids. After twofold dilution with ultrapure water, internal standards (AA-d8, 15-HETE-d8, 14,15-EET-d11, LTB_4_-d4, PGE_2_-d4, and LTD_4_-d5) were added. The samples were injected into an ion exchange column (MonoSpin Ion Exchange Column C18-AX, GL Sciences) conditioned with methanol and ultrapure water. After washing with ultrapure water and 50% methanol, the lipids were eluted with 90% methanol containing 2% acetic acid. External standards (PGB_2_-d4 and 8-iso-PGF_2a_-d4) were added to the extract.

### HPLC fractionation

The methyl formate fraction was subjected to HPLC fractionation using a modified version of our previous method for separating oxidized fatty acids^41^. Specifically, the methyl formate fraction was diluted twofold with ultrapure water and injected. The separation process was performed using an HPLC system equipped with a C18 column (XBridge BEH C_18_ Column, 130 Å, 5 µm, 4.6 mm × 100 mm, Waters), with a mobile phase consisting of a mixture of water/methanol/acetic acid (50:50:0.01, v/v/v, mobile phase A) and methanol (mobile phase B). The separation was performed at a flow rate of 0.7 mL/min for 5 min using 90% mobile phase A, followed by a gradient to 100% mobile phase B over a period of 25 min, and then held for an additional 10 min.

### Lipidomics analysis

LC–MS/MS-based lipidomics was performed to measure the composition and concentration of fatty acid-derived lipophilic metabolites as previously reported^30, 31^. Briefly, the extracted lipids were analyzed by LC–MS/MS using a triple quadrupole linear ion trap mass spectrometer (QTRAP5500; AB SCIEX) equipped with an Acquity UPLC BEH C_18_ column (1.7 µm, 1.0 mm × 150 mm; Waters). LC separation was performed with gradient elution of mobile phases composed of mobile phase A [water/acetic acid (100:0.1, v/v)] and mobile phase B [acetonitrile/methanol (4:1, v/v)]. The initial mobile phase A/B (73:27) was allowed to flow for 5 min, then linearly converted to mobile phase A/B (30:70) for 15 min, to mobile phase A/B (20:80) for 25 min, held for 8 min, linearly converted to mobile phase A/B (5:95) for 35 min, held for 4 min, ramped to mobile phase A/B (0:100), and held for 40 min. The flow rate was 0.05 mL/min up to 30 min, 0.08 mL/min from 30 to 33 min, and 0.1 mL/min from 33 to 40 min. The MS/MS analysis was performed in negative mode, and fatty acid metabolites were measured by multiple reaction monitoring.

### Preparation of lymphocytes

Small intestinal and colonic lamina propria lymphocytes were isolated as described previously^7^. In brief, intestinal tissues were incubated in HBSS (Nacalai tesque) containing 1 mM dithiothreitol and 20 mM EDTA at 37 °C for 20 min. The tissues were minced and then incubated in collagenase solution containing 0.125 mg/mL collagenase (Wako Pure Chemical Industries), 0.5 mg/mL DNase I (Roche Diagnostics), 2% FBS, 100 U/mL penicillin, 100 μg /mL streptomycin, and 12.5 mM HEPES, in RPMI 1640 medium (Nacalai tesque) at 37 °C for 30 min. Then, the cell suspensions were washed with 2% FBS in PBS and were subjected to Percoll (Cytiva) gradient separation. The spleen and MLN were mechanically crushed into single-cell suspensions.

### DSS colitis

Mice were received 2% DSS (molecular weight: 36,000–50,000; MP Biomedicals, Santa Ana, CA, USA) in their drinking water for up to 7 days. Body weight and DAI scores were monitored daily during the experimental period for DSS colitis. The DAI scores were assessed based on body weight loss (0: no loss; 1: 1–5% loss; 2: 5–10% loss; 2: 5–10% loss), stool consistency (0: formed; 1: mild soft; 2: very soft; and 3: watery stools), and stool bleeding (0: normal color stool with negative hemoccult analysis; 1: brown-colored stool with positive hemoccult analysis; 2: reddish-colored stool with traces of blood; and 3: visible rectal bleeding). The presence of occult blood was tested using ColoScreen-ES (Helena Laboratories, Beaumont, TX, USA).

### Cell isolation and flow cytometry

Naïve T cells were isolated from the spleens of C57BL/6J mice according to a previously described method^7^. CD4^+^ T cells were enriched by negative selection with the IMag Cell Separation System (BD Biosciences) using a mixture of biotinylated mAbs against CD8a, CD11b, CD11c, B220, Gr-1, and TER-119 and Streptavidin Particle Plus-DM (BD Biosciences). Naïve T cells (CD45^+^ CD4^+^ CD62^high^ CD44^low^ CD25^-^ NK1.1^-^) were sorted using a FACSAria III cell sorter from the enriched CD4^+^ fraction (Supplemental Fig. 1B). DCs were enriched from the spleen of CD57BL/6J mice by a positive selection method using the IMag Cell Separation System (BD Biosciences) with a mixture of PE-conjugated mAbs against CD11c and anti-R-phycoerythrin (PE) particles (BD Biosciences). The enriched CD11c^+^ fraction was subjected to cell sorting using a FACSAria III to isolate CD45^+^ CD11c^+^ MHC class II ^+^ DCs (Supplemental Fig. 1C). Cultured cells were incubated with anti-CD16/32 (FcγR) antibodies to block non-specific reactions and stained with specific antibodies. Cells were fixed for intracellular transcription factor staining using the eBioscience Foxp3/Transcription Factor Staining Buffer Set (Thermo Fisher Scientific) for 45 min. After fixation, transcription factors were stained with specific antibodies. Cells were analyzed using a FACS LSR II flow cytometer (BD Biosciences) (Supplemental Fig. 1D).

The antibodies used for flow cytometry were as follows: anti-CD16/32 (93), biotinylated anti-mouse CD8α (53-6.7), biotinylated anti-mouse CD11b (M1/70), biotinylated anti-mouse CD11c (N418), biotinylated anti-mouse/human CD45R/B220 (RA3-6B2), biotinylated anti-Ly6G/Ly-6C (Gr-1) (RB6-8C5), biotinylated anti-TER-119/Erythroid cells (TER-119), BV510 anti-mouse CD45 (30-F11), Alexa488 anti-mouse NK-1.1 (PK136), APC anti-mouse/human CD44 (IM7), PerCP-Cy5.5 anti-mouse TCRβ (H57-597), BV510 anti-mouse CD45 (30-F11), and BV421 anti-mouse CD4 (RM4-5), which were purchased from BioLegend; eF450 anti-mouse CD62L (MEL-14), PE-Cy7 anti-mouse CD25 (PC61.5), PE anti-CD11c (N418), eF450 anti-MHC class II (M5/114.15.2), eF780 anti-mouse CD4 (RM4-5), and eF660 anti-Foxp3 (FJK-16s), which were purchased from Thermo Fisher Scientific. For intracellular staining, dead cells were detected using the 7-AAD Viability Staining Solution (BioLegend) or Fixable Viability Stain 780 (BD Biosciences).

### In vitro cultures

For naïve T cell and DC co-cultures, naive CD4^+^ T cells (5 × 10^5^ cells/mL) and DCs (5 × 10^5^ cells/mL) were co-cultured in 96-well U-bottom plates and stimulated with soluble anti-CD3ε mAb (5 μg/mL) supplemented with 0.1 ng/mL TGF-β1 and 10 ng/mL IL-2 (BioLegend) for 3 days. For naïve T cell monocultures, naive CD4^+^ T cells (5 × 10^5^ cells/mL) were cultured in 96-well flat-bottom high-binding plates (Corning). The cells were stimulated with immobilized anti-CD3ε mAb (5 μg/mL) and soluble anti-CD28 mAb (5 μg/mL) supplemented with 0.1 ng/mL TGF-β1 and 10 ng/mL IL-2 (BioLegend) for 3 days. LA, 10,13(OH)18:0, 17,18-DiHETE, 9,10-EpOME, 9,10-DiHOME, and 9,10-EpOME were purchased from Cayman Chemical Company and stored at -30 °C in methyl acetate before use. For all experiments, the methyl acetate was removed under a nitrogen steam. The compound was resuspended in TexMACS medium (Miltenyi Biotec) containing 20 µM GlutaMAX (Thermo Fisher Scientific), 55 µM 2-mercaptoethanol, 10 mM HEPES, 100 U/mL penicillin, and 100 µg/mL streptomycin, or RPMI 1640 medium containing 10% FBS, 20 µM GlutaMAX (Thermo Fisher Scientific), 55 µM 2-mercaptoethanol (Thermo Fisher Scientific), 10 mM HEPES, 100 U/mL penicillin, and 100 µg/mL streptomycin. The methyl formate fraction dissolved in ethanol was diluted in the culture medium at the same ethanol concentration (0.5%) under all dilution conditions.

### Preparation of BMDCs

BMDCs were prepared as described previously^42^. Bone marrow cells were isolated from the femurs and tibias of mice, and 2 × 10^6^ cells/mL of bone marrow cells were cultured in RPMI 1640 medium containing 10% FBS, 20 µM GlutaMAX I (Thermo Fisher Scientific), 55 µM 2-mercaptoethanol (Thermo Fisher Scientific), 10 mM HEPES, 100 U/mL penicillin, and 100 µg/mL streptomycin supplemented with 10 ng/mL GM-CSF, 10 ng/mL IL-4, and 5 ng/mL Flt-3L for 6 days. The medium was changed on day 3. The BMDCs were stimulated with lipopolysaccharide (LPS) (100 ng/mL; Sigma, St. Louis, MO, USA) in the presence or absence of 9,10-DiHOME for 24 h.

### Ex vivo fermentation analysis

Ex vivo fermentation was performed as previously described^26, 43^. Briefly, cecal contents were obtained from 9-week-old Wistar rats and diluted 50-fold with saline. Then, 150 mL of diluted cecal contents were incubated in a jar fermenter at 37 °C under an anaerobic atmosphere with gentle stirring and pH-controlled (pH > 5.2) conditions. After overnight preincubation, LA (final concentration, 20 µM) was added to the culture supernatant. Four microliters of the culture supernatant was collected at 0 and 48 h after supplementation of LA to monitor the organic acid content.

### Statistical analysis

Values are expressed as mean ± SD. Differences between the mean values of each group and vehicle or untreated group were analyzed by unpaired *t*-tests or one-way analysis of variance, followed by Dunnett's test. GraphPad version 7 or 8 was used for all statistical analyses. Differences were considered statistically significant when *P* values were < 0.05.

### Ethics declarations

All animal experiments were approved by the Animal Research Committee of Keio University and Obihiro University of Agriculture and Veterinary Medicine, and were performed in accordance with relevant guidelines and regulations (Institutional Guidelines on Animal Experimentation at Keio University and Obihiro University of Agriculture and Veterinary Medicine). This study is reported in accordance with ARRIVE guidelines.

## Supplementary Information

Supplementary Figures.

## Data Availability

The data in this study are available from the corresponding author K.H., upon reasonable request.
