# Supplementary material for: Gut microbiota-derived lipid metabolites facilitate regulatory T cell differentiation
Source: Sci Rep. 2023 Jun 1;13:8903. doi: 10.1038/s41598-023-35097-5 (PMC10235104; doi:10.1038/s41598-023-35097-5)
Supplement: Supplementary file 1 — Supplementary Figures. [file 41598_2023_35097_MOESM1_ESM.pdf]

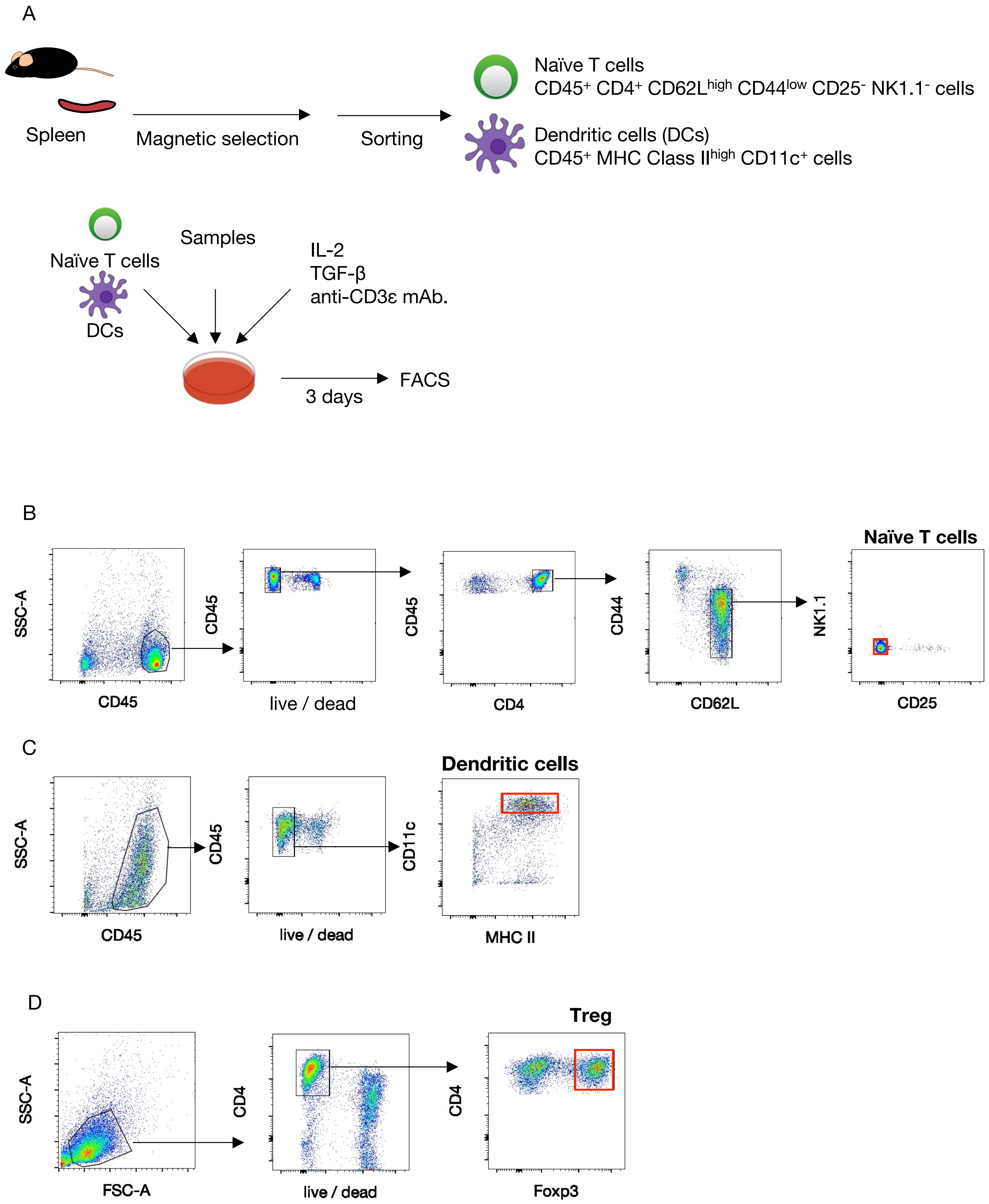


**Supplemental figure 1. Evaluation of Treg-inducing ability of lipophilic metabolites *in vitro*.**
(A) Splenic naive T cells and DCs were isolated by magnetic selection and FACS, and co-cultured for 3 days in Treg-inducing conditions. Gating strategy of naïve T cells (B), DCs (C) and Treg cells (D).

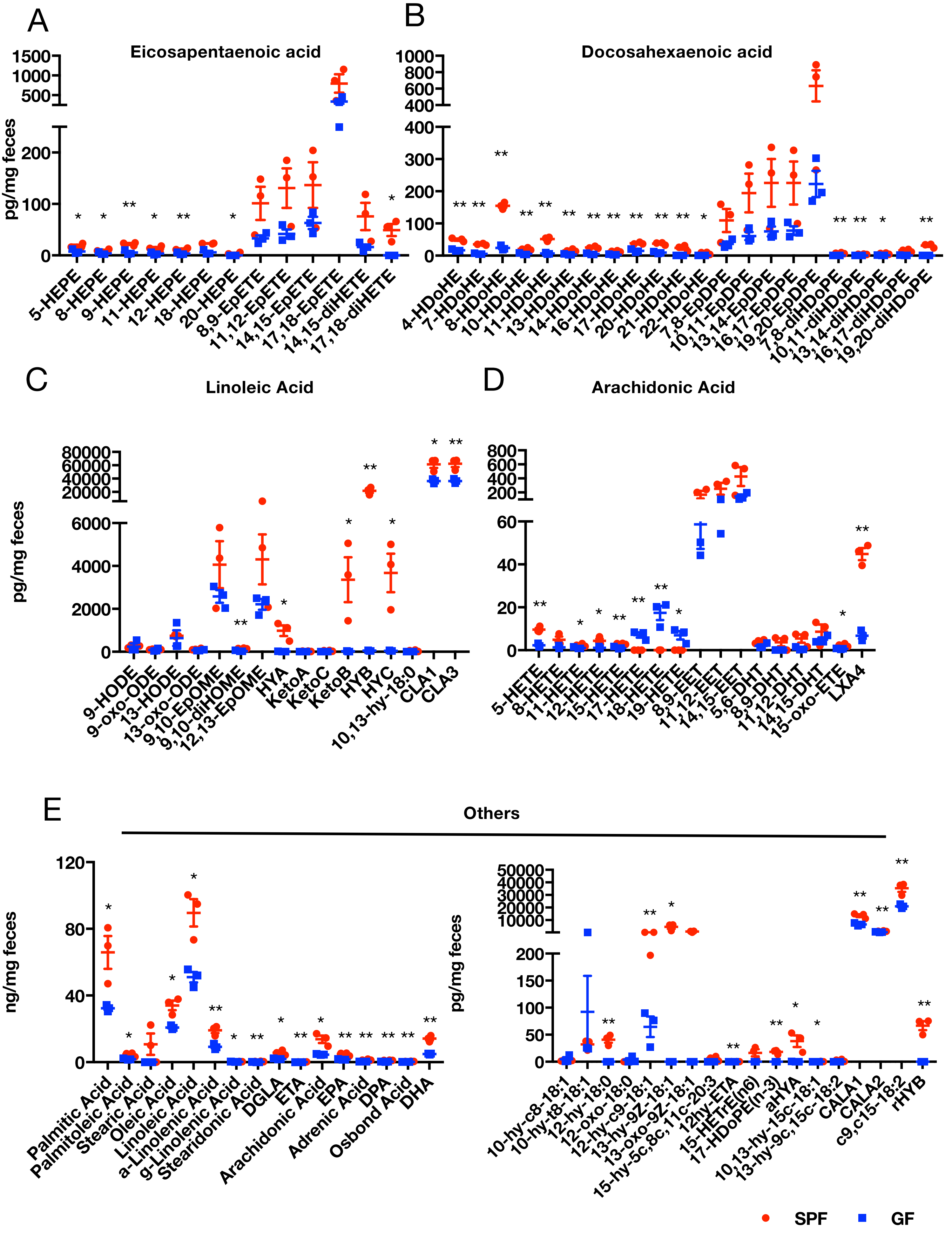


**Supplemental figure 2. Lipidomic analysis of oxidized fatty acids in fecal-derived methyl formate fractions.** Lipids in the methyl formate fraction were extracted from SPF and GF mouse feces by solid phase extraction and (A) eicosapentaenoic acid- (B) docosahexaenoic acid- (C) linoleic acid- (D) arachidonic acid derived metabolites and (E) others were analyzed by LC-MS/MS lipidomics.

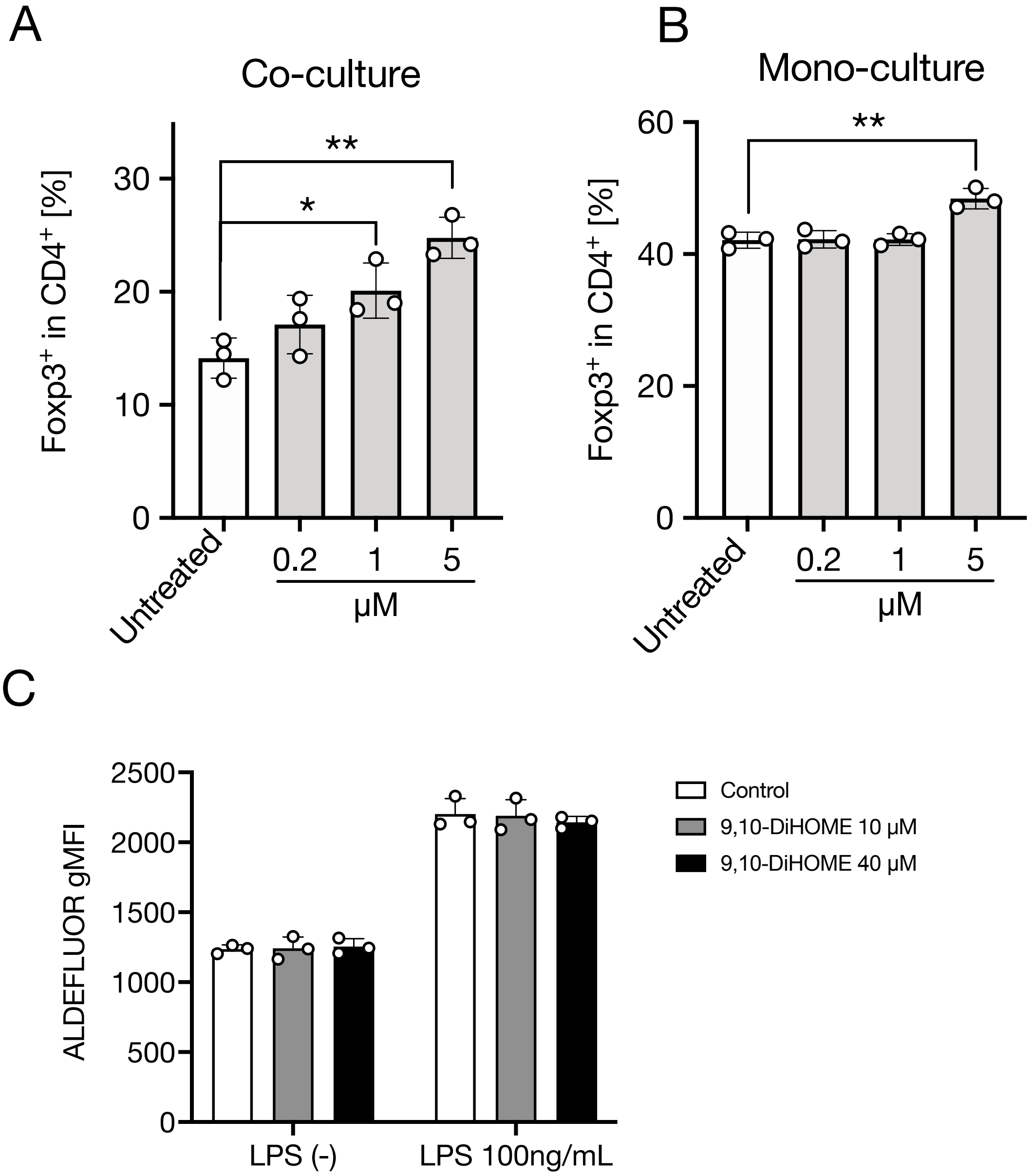


**Supplemental figure 3. 9,10-DiHOME induces Treg differentiation in DCs dependent pathway**.
(A, B) Treg-inducing effect of 9,10-DiHOME was analyzed in the presence (A) or absence (B) of splenic DCs.
(C) gMFI of ALDEFLUOR$^{+}$ BMDCs treated by 9,10-DiHOME and LPS for 24 h.
The data represent the mean ± SD. n = 3, **P* < 0.05, ***P* < 0.01. *P* values determined by Dunnet's test (A, B), Two-way ANOVA followed by Tukey's test (C)

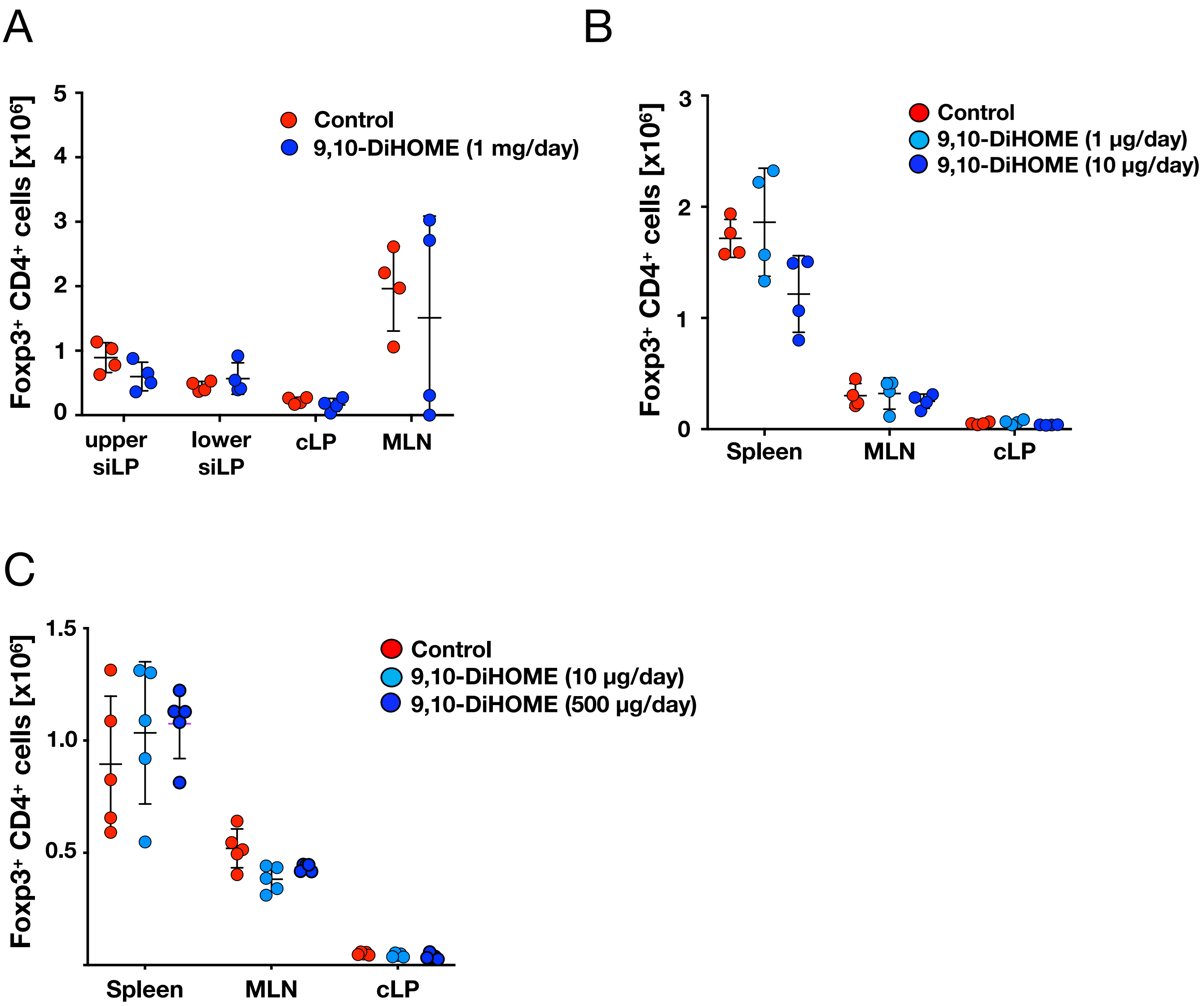


**Supplemental figure 4. The effect of 9,10-DiHOME administration on Treg cells *in vivo*.**
Three-week-old mice received 9,10-DiHOME every day for 3 weeks by intragastric administration (A), intrarectal administration (B), or intraperitoneal injection (C). The number of Treg cells in the small intestinal lamina propria (siLP) and colonic lamina propria (cLP), spleen, and MLN was analyzed by flowcytometry.
